# Miscellaneous—Special

**Published:** 1893-12-23

**Authors:** 


### MISCELLANEOUS.—SPECIAL.

**Cancer Hospital (Free),** Fulham Road, S.W.—We desire to draw the attention of our readers to the claims of this valuable special hospital on their support. Founded in 1851, its work has gone on increasing year by year. During last year there were 811 new in-patients and 1,289 new out-patients, whilst some 9,279 visits of new and old out-patients were made during the same period. Within the last twelve months several important additions have been made to this hospital, involving a considerable outlay, including the erection of a chapel, relaying the floors of four wards with teak, building a kitchen, nurses' bed-room and bath-room for the isolation wards, providing additional sleeping accommodation for the nurses, and making certain sanitary alterations in the hospital. Unfortunately the continued increase in the number of patients is a source of corresponding increase in the expenditure. The secretary, Mr. W. H. Hughes, will be glad to receive contributions to meet this, and we would strongly urge those benevolent persons who may be disposed to help in this good work to send their donations to him.

**Central London Throat and Ear Hospital,** Gray's Inn Road, W.C.—The hospital was established twenty years ago for the treatment of poor patients suffering from diseases of the throat, nose, and ear. It is situated in a very poor and populous district, while its contiguity to the various railway termini at King's Cross renders it easily accessible for patients from all parts of London, the suburbs, and the country. The hospital has no endowment whatever, and is dependent entirely upon voluntary contributions. Secretary, Mr. R. Kershaw; Matron, Miss M. Danter.

**Dental Hospital of London,** Leicester Square.—The Dental Hospital is contemplating moving to a new site as soon as the money required (probably about £40,000) is forthcoming. Promises have already been received to the amount of about £10,000, and as soon as £20,000 is collected to purchase a site offered adjoining the present hospital, a new building will be started. The present hospital in Leicester Square, which has for many years so ably fulfilled its duties, has, with the increasing number of patients and students, become too small for present requirements. From the fact that this hospital is for the treatment of the teeth only, it does not, perhaps, receive that support from the public it deserves. To carry on a dental hospital the annual expenditure is necessarily large for the materials used, and notwithstanding the fact that the teeth are considered of trifling importance, there is no doubt they are serious factors in the general welfare of the public. Secretary, Mr. J. Francis Pink.

**Hospital for Diseases of the Throat,** Golden Square, Regent Street, W.—The committee appeal for donations and subscriptions towards the maintenance of the children's ward, and they earnestly hope that some portion of the charitable gifts of the season will be devoted to this object. The special ward recently opened for children is kept constantly full, and a very large number of children are awaiting their turn for admission. Secretary, W. Holt; Matron, Miss M. B. Mackey.

**Lock Hospital and Home,** Harrow Road, W.—Rescue work, as well as the cure of the patients who present themselves for admission, is the aim of this institution, and the good work has only to be known to meet with the support it deserves. Considerably more than one-fourth of those that pass through the hospital are reclaimed from their evil lives, which points to a great work. A debt of £5,000 has to be cleared off, and £1,500 is required in fresh annual subscriptions to meet the regular requirements of the institution. Secretary, Mr. A. W. Cruikshank; Matron of hospital, Mrs. Kydd; Matron of home, Miss J. McNiven.

**Royal London Ophthalmic Hospital,** Moorfields, E.C.—An urgent appeal is made by the Board of Management for funds in support of this institution. The expenditure amounts annually to over £6,000, whereas the certain income is under £2,000. The patients numbered in 1892 a total of 28,462; of these 1,967 were in-patients, and the total attendances of the out-patients was 112,475. All patients are admitted free, and without letters of recommendation. Bankers: Williams, Deacon, and Co., 20, Birchin Lane, E.C.; Secretary, Mr. Robert J. Newstead; Matron, Miss Nichol.

**National Dental,** Great Portland Street.—The fine new building, at the corner of Great Portland Street and Devonshire Street, which takes the place of the old house, 149, Great Portland Street, is now in full working order. The Duke of York has become the new President, and Mr. Charles Hoare, Treasurer. Secretary, E. Almack.

